# Every woman in the world must have respectful care during childbirth: a reflection

**DOI:** 10.1186/s12978-020-0855-x

**Published:** 2020-01-22

**Authors:** José M. Belizán, Suellen Miller, Caitlin Williams, Verónica Pingray

**Affiliations:** 1 Department of Mother and Child Health Research, Institute for Clinical Effectiveness and Health Policy (IECS-CONICET), Buenos Aires, Argentina; 2 Safe Motherhood Program, University of California, San Francisco, USA; 3 Department of Maternal & Child Health Gillings School of Global Public Health, University of North Carolina at Chapel Hill, Chapel Hill, USA

Every woman has the right to the highest attainable standard of health, which includes the right to respectful maternity care [1]. We—as pregnant and birthing individuals and the care providers, public health professionals, and researchers serving them—know instinctually what constitutes dignified treatment. Yet the systems and structures within which we birth and work are not designed to ensure respectful, evidence-based care.

To help the reader see this more clearly, we invite you to do a thought experiment. Imagine you are a woman in labour. You come to a facility in order to receive quality obstetric care. What type of treatment would you expect? Timely attention? Clear and detailed information from a caring health provider about what to expect and why? Recognition of your role as an active decision-maker and protagonist in your own birthing experience, with the choice to consent to or refuse any procedures once you understand them and their implications? Perhaps having a chosen companion with you at all times or deciding on birth position(s) based on your own comfort? Or maybe having the privacy to experience your newborn’s first hours without sharing a bed with a stranger? What other expectations would you have? Viewed in this way, conceptualizing dignified treatment is simple.

Yet such timely, respectful and consensual obstetric care is not the norm in many healthcare settings across the globe. There is a wide-spread belief that ensuring safe birth requires placing the needs and priorities of health providers over those of birthing women. This sets up and perpetuates a power imbalance, privileging providers and contributing to obstetric violence. The power imbalance between women and providers is echoed and exacerbated by similar power dynamics between providers (across cadre and seniority) that can produce counterproductive and even toxic interactions between members of the care team, undermining quality of care and contributing to provider burnout [2].

It is critical that we all reflect individually on these issues, because *we*—collectively as society—create the written and unwritten rules and norms that govern institutions (be they health facilities; schools of medicine, midwifery, and nursing; or safe motherhood initiatives); therefore, *we* can also be the driving force to change them. Clear your mind of the idea that the power dynamics in the institutions under which we live are natural. They are not; and making such a dangerous mistake misleads us into believing that we are exempt from acting.

In the Millennium Development Goal-era push to reduce maternal and newborn mortality and morbidity, strong recommendations and actions were taken to reduce home births and encourage women to instead give birth in health facilities. Unfortunately, there was a large missing element. While we have seen rates of facility delivery increase dramatically, we have not seen a concomitant improvement in women’s experience of childbirth. The shift from birthing at home to birthing in facilities helped increase access to life-saving care for complications, but also introduced new challenges, including overcrowding of facilities, an excess of procedures, and over-medicalization of birth. In fact, we now know that facility birth does not on its own lead to improved outcomes; these rely on quality, respectful, evidence-based care [3].

The foundations for the contemporary focus on respectful care were laid in Latin America in the 1970s and 1980s. The publication (in Spanish) of *Physiological and Psychological Bases for the Humanized Management of Natural Birth* by Roberto Caldeyro-Barcia in the Latin American Centre for Perinatology, along with the jointly-led WHO and PAHO 1985 Fortaleza Declaration foregrounded the importance of dignified treatment [4, 5]. Subsequent work focused this new global attention on centring maternal satisfaction with the birthing process, uplifting positive traditional and indigenous practices, and identifying the health system conditions that contribute to mistreatment [6–8].

Within the last decade, respectful care in childbirth has garnered renewed attention, this time among a broader range of global health actors. For example, in Latin America, advocates pushed for legal frameworks addressing the issue [9]. The articles published in the Respectful Care series of this Journal reflect this, documenting the lack of dignified treatment in many countries: Tunisia, Nigeria, Guinea, Brazil, Tanzania, Ethiopia, India, South Africa, the United States, and among Romani women in Europe [10–19]. Yet today we find ourselves at an inflection point: it is time for us to move from merely documenting the problem towards engaging women, their families, and communities in jointly designing and testing effective, meaningful interventions.

It is imperative that we provide the most respectful, humane, careful, friendly, effective, evidence-based childbirth care in our health facilities. At *Reproductive Health*, we are eager to receive and publish manuscripts to help achieve such care. Contributions from women and their families would be greatly appreciated, such as submissions describing their vision for respectful care and experiences, as well as offering suggestions for increasing respectful care in facilities. We welcome manuscripts from health facility staff from all levels—administration, nursing, midwifery, medicine, program managers, and decision makers—as well as manuscripts from social scientists on interventions to help providers change their attitudes and practices, and to encourage communities to demand their right to respectful care. We also seek articles from human rights activists and policymakers on actions to protect the right to respectful care during childbirth. As is stated in one of the articles published in the Journal’s Respectful Care Series: *“The compassion and evidence based medicine agenda in healthcare is interconnected with human rights in healthcare, feeding into the principles of decision making and patient centred care”* ( [20], abstract)*.*

As disrespect and abuse in childbirth has gained public traction, an interesting global semiotic discussion has arisen on the terminology that best defines it. For this series, we have selected the use of Respectful Care over the negative terms (“disrespect and abuse”, “mistreatment during childbirth”, or “obstetric violence”), in order to focus on the positive aspects of care and caring as a broader concept that encompasses all of what pregnant and childbearing people and their families deserve, and not just the absence of mistreatment [21]. By employing the term *respectful care,* we intend to set a shared goal for all actors, from lay individuals and health providers to researchers and policymakers. We expect that by joining efforts we can achieve a change in the delivery of dignified obstetric care.

The Chilean writer, Isabel Allende, thoroughly narrates in her book *De Amor y de Sombra*, Digna’s first experience giving birth in a hospital, after having had five home deliveries:

“*Digna had gone to Los Riscos Hospital, where she felt she had been treated worse than a criminal. When she entered a numbered band was strapped around her wrist, they shaved her private parts, bathed her with cold water and antiseptic, (…) and placed her beside a woman in the same condition on a bed without sheets. After poking around, without her permission, in all her bodily orifices, they made her give birth beneath a bright lamp in full view of anyone who might happen by. She bore it all without a sigh, but when she left that place carrying a baby that was not hers in her arms and with her unmentionable places painted red like a flag, she swore that for the rest of her life she would never again set foot in a hospital.”* (Translation by Margaret Sayer Peden) ( [22], p., 20).

In order to continue efforts to improve maternal and newborn health, it is our responsibility to ensure that no woman in the world leaves a health facility feeling like Digna. We call on all readers to work together to achieve universal respectful care for every woman, everywhere.
