# Correction to: Toda mujer en el mundo debe tener un cuidado respetuoso durante el parto: una reflexión

**DOI:** 10.1186/s12978-020-00950-7

**Published:** 2020-07-23

**Authors:** José M. Belizán, Suellen Miller, Caitlin Williams, Verónica Pingray

**Affiliations:** 1 Department of Mother and Child Health Research, Institute for Clinical Effectiveness and Health Policy (IECS-CONICET), Buenos Aires, Argentina; 2 Safe Motherhood Program, University of California, San Francisco, USA; 3 Department of Maternal & Child Health Gillings School of Global Public Health, University of North Carolina at Chapel Hill, Chapel Hill, USA

**Correction to: Reproductive Health (2020) 17:7**

**https://doi.org/10.1186/s12978-020-0855-x**

Toda mujer tiene derecho al más alto nivel posible de salud, lo que incluye el derecho a una atención respetuosa de la maternidad [1]. Las mujeres embarazadas y las que dan a luz, así como los proveedores de atención médica, los profesionales de la salud pública y los investigadores que les prestan servicios, saben instintivamente lo que constituye un trato digno. Sin embargo, los sistemas y estructuras dentro de los cuales nacemos y trabajamos no están diseñados para asegurar una atención respetuosa y basada en la evidencia.

Para ayudar al lector a ver esto más claramente, le invitamos a hacer un experimento de pensamiento. Imagine que es una mujer en trabajo de parto. Viene a un centro para recibir atención obstétrica de calidad. ¿Qué tipo de tratamiento esperaría? ¿Atención oportuna? ¿Información clara y detallada de un proveedor de salud sobre qué esperar y por qué? ¿Reconocimiento de su papel como una activa tomadora de decisiones y protagonista en su propia experiencia de parto, con la opción de consentir o rechazar cualquier procedimiento una vez que lo entienda y sus implicaciones? ¿Quizás tener un acompañante elegido por usted en todo momento o decidir la posición o posiciones en el parto en función de su propia comodidad? ¿O quizás tener la privacidad de experimentar las primeras horas de su recién nacido sin compartir la cama con un extraño? ¿Qué otras expectativas tendría usted? Visto de esta manera, conceptualizar un tratamiento digno es simple.

Sin embargo, este tipo de atención obstétrica oportuna, respetuosa y consensuada no es la norma en muchos centros de atención médica en todo el mundo. Existe la creencia generalizada de que para garantizar un parto seguro es necesario situar las necesidades y prioridades de los proveedores de salud por encima de las de las mujeres que dan a luz. Esto crea y perpetúa un desequilibrio de poder, privilegiando a los proveedores y contribuyendo a la violencia obstétrica. El desequilibrio de poder entre las mujeres y los proveedores se ve reflejado y exacerbado por dinámicas de poder similares entre los proveedores (en los cuadros y en la antigüedad) que pueden producir interacciones contraproducentes e incluso tóxicas entre los miembros del equipo de atención, lo que socava la calidad de la atención y contribuye al agotamiento del proveedor [2].

Es fundamental que todos reflexionemos individualmente sobre estas cuestiones, porque nosotros, colectivamente como sociedad, creamos las reglas y normas escritas y no escritas que rigen las instituciones (ya sean centros de salud, facultades de medicina, partería y enfermería o iniciativas de maternidad segura); por lo tanto, también podemos ser la fuerza motriz para cambiarlas. Despeja tu mente de la idea de que la dinámica de poder en las instituciones bajo las que vivimos es natural. No lo son; y cometer un error tan peligroso nos hace creer que estamos exentos de actuar.

En el impulso de la era de los Objetivos de Desarrollo del Milenio para reducir la mortalidad y la morbilidad materna y neonatal, se formularon recomendaciones y medidas enérgicas para reducir los partos en el hogar y alentar a las mujeres a dar a luz en centros de salud. Lamentablemente, había un gran elemento que faltaba. Si bien hemos visto que las tasas de partos en establecimientos sanitarios han aumentado drásticamente, no hemos visto una mejora concomitante en la experiencia de las mujeres en los partos. El paso de los partos en el hogar a los partos en centros de salud ayudó a aumentar el acceso a la atención que salva vidas en caso de complicaciones, pero también introdujo nuevos desafíos, como el hacinamiento en los hospitales, el exceso de procedimientos y la sobremedicalización de los partos. De hecho, ahora sabemos que los partos en establecimientos no conducen por sí solos a mejores resultados; éstos dependen de una atención de calidad, respetuosa y basada en evidencias [3].

Los cimientos del enfoque contemporáneo de la atención respetuosa se establecieron en América Latina en los años setenta y ochenta. La publicación (en español) de Bases fisiológicas y psicológicas para el manejo humanizado del parto natural de Roberto Caldeyro-Barcia en el Centro Latinoamericano de Perinatología, junto con la Declaración de Fortaleza de 1985 de la OMS y la OPS, puso de relieve la importancia del trato digno [4, 5]. La labor posterior centró esta nueva atención mundial en centrar la satisfacción materna en el proceso de parto, elevar las prácticas tradicionales e indígenas positivas e identificar las condiciones del sistema de salud que contribuyen al maltrato [6–8].

En el último decenio, la atención respetuosa en el parto ha suscitado una atención renovada, esta vez entre una gama más amplia de agentes de salud mundiales. Por ejemplo, en América Latina, los defensores abogaron para que se establecieran marcos jurídicos que abordaran la cuestión [9]. Los artículos publicados en la serie “Respectful Care” de esta Revista lo reflejan, documentando la falta de un trato digno en muchos países: Túnez, Nigeria, Guinea, Brasil, Tanzanía, Etiopía, India, Sudáfrica, Estados Unidos y entre las mujeres romaníes de Europa [10–19]. Sin embargo, hoy nos encontramos en un punto de inflexión: ha llegado el momento de pasar de la mera documentación del problema a la participación de las mujeres, sus familias y comunidades en el diseño y ensayo conjunto de intervenciones eficaces y significativas.

Es imperativo que proveamos el cuidado de parto más respetuoso, humano, cuidadoso, amigable, efectivo y basado en evidencia en nuestras instalaciones de salud. En la revista Reproductive Health, estamos entusiastas en recibir y publicar manuscritos para ayudar a lograr dicha atención. Se agradecerán enormemente las contribuciones de las mujeres y sus familias, que describan su visión de una atención respetuosa y sus experiencias, así como las sugerencias para mejorar la atención respetuosa en los establecimientos de atención en salud. Acogeremos con agrado manuscritos del personal de los centros de salud de todos los niveles -administración, enfermería, obstetricia, medicina, directores de programas y responsables de la toma de decisiones-, así como los manuscritos de científicos sociales sobre intervenciones para ayudar a los proveedores a cambiar sus actitudes y prácticas, y para alentar a las comunidades a exigir su derecho a una atención respetuosa. También apreciaremos manuscritos de activistas de derechos humanos y responsables de la formulación de políticas sobre acciones para proteger el derecho a una atención respetuosa durante el parto. Como se afirma en uno de los artículos publicados en la Serie de Cuidados Respetuosos de la Revista: “La agenda de la medicina basada en el trato respetuoso y en la evidencia en el cuidado de la salud está interconectada con los derechos humanos, contribuyendo con los principios de la toma de decisiones y el cuidado centrado en el paciente” ([20], resumen).

A medida que la falta de respeto y el abuso en el parto ha ido ganando terreno en la opinión pública, ha surgido un interesante debate semiótico global sobre la terminología que mejor lo define. Para esta serie, hemos seleccionado el uso de Respectful Care por encima de los términos negativos (“falta de respeto y abuso”, “maltrato durante el parto” o “violencia obstétrica”), con el fin de centrarnos en los aspectos positivos de la atención y el cuidado como un concepto más amplio que abarca todo lo que merecen las personas embarazadas y en edad fértil y sus familias, y no sólo la ausencia de maltrato [21]. Al emplear el término cuidado respetuoso, nos proponemos establecer un objetivo compartido por todos los actores, desde los legos y los proveedores de salud hasta los investigadores y los encargados de formular políticas. Esperamos que uniendo esfuerzos podamos lograr un cambio en la prestación de una atención obstétrica digna.

La escritora chilena, Isabel Allende, relata con detalle en su libro De Amor y de Sombra, la primera experiencia de Digna al dar a luz en un hospital, después de haber tenido cinco partos en casa:

““Digna acudió al Hospital de los Riscos donde se sintió tratada peor que un condenado. Al entrar le pusieron un parche con un número en la muñeca, le afeitaron sus partes pudorosas, la bañaron con agua fría y desinfectante y le colocaron en una cama con otra mujer en sus mismas condiciones. Después de hurgar en todos los orificios de su cuerpo sin pedirle permiso, la hicieron dar a luz debajo de una lámpara a la vista de quien quisiera curiosear. Todo lo soportó con un suspiro pero cuando salió de allí con sus vergüenzas pintadas de rojo como una bandera, juró no velver a poner los pies en un hospital en los días de su vida” ([22], p., 20).

A fin de continuar los esfuerzos por mejorar la salud materna y neonatal, es nuestra responsabilidad asegurarnos de que ninguna mujer del mundo abandone un centro de salud sintiéndose como Digna. Hacemos un llamamiento a todos los lectores para que trabajemos juntos a fin de lograr una atención universal respetuosa de todas las mujeres, en todas partes.
